# Chironomid larvae, important hosts facilitating the persistence of *Vibrio cholerae* in water reservoirs of a cholera-endemic area of north Cameroon

**DOI:** 10.11604/pamj.2025.52.16.43072

**Published:** 2025-09-15

**Authors:** Roméo Wakayansam Bouba, Moussa Djaouda, Pagoui Ehbiakbo, Eric Moïse Bakwo Fils, Céline Nguefeu Nkenfou

**Affiliations:** 1 Department of Biological Sciences, Faculty of Science, University of Maroua, PO Box 814, Maroua, Cameroon,; 2 Department of Life and Earth Sciences, Higher Teachers´ Training College, University of Maroua, PO Box 55, Maroua, Cameroon,; 3 Institue of Fine Arts and Innovation, University of Garoua, PO Box 346, Garoua, Cameroon,; 4 Department of Biological Sciences, Higher Teachers´ Training College, University of Yaoundé 1, P.O. Box 47, Yaoundé, Cameroon,; 5 Chantal Biya´s International Reference Centre (CBIRC), Centre for HIV/AIDS Prevention and Management, PO Box 3077, Yaoundé, Cameroon

**Keywords:** Chironomids, *Vibrio cholerae*, waters, north Cameroon

## Abstract

**Introduction:**

chironomid larvae are present in aquatic systems, including freshwater, and often are important hosts for Vibrio cholerae. Chironomid larvae interact with V. cholera and may shield the bacterium from environmental stressors, resulting in its dissemination. This study aimed to determine the role of chironomid larvae in the persistence of V. cholera in the water reservoirs of a cholera-endemic area of Cameroon.

**Methods:**

chironomid larvae and water samples were collected from scoop holes of the Kaliao and Mizao temporary streams in Maroua during the cholera inter-epidemic (dry) season. V. cholera was isolated from chironomid larvae and water samples on thiosulfate citrate bile salt sucrose agar and biochemically identified. Water temperature, salinity, electrical conductivity, total dissolved solids, oxygen saturation, dissolved oxygen, and hardness (including calcium and magnesium) were analyzed for their influence on V. cholerae-larvae interactions.

**Results:**

Vibrio cholera was isolated in 45.45% of cases (95% CI: 32.5-58.9) from chironomid larvae. In contrast, 27.72% of water samples without larvae tested positive for V. cholera (95% CI: 18.4-38.6), only 4.54% of samples simultaneously harbored V. cholera in both larvae and water (95% CI: 1.2-11.3). The probability of detecting V. cholera was significantly higher in chironomid larvae than in water (p < 0.05), suggesting a strong ecological association. Furthermore, water samples with higher dissolved oxygen saturation (up to 91%) and elevated magnesium concentrations (up to 133.65 mg/L) were consistently V. cholerae-negative, suggesting a potential inverse relationship between these parameters and bacterial presence.

**Conclusion:**

the study highlights chironomid larvae as potential reservoirs for V. cholerae, emphasizing the need to monitor both larval habitats and water quality for better cholera surveillance and control in endemic regions.

## Introduction

At the beginning of the 21^st^ century, infectious diseases were responsible for almost 25% of human mortality, with over 14 million deaths annually [1]. Infectious diseases represent a major public health issue and are often linked to living conditions [2]. Worldwide, many infectious diseases in humans and animals are waterborne [3]. Among these diseases, cholera is one of the most devastating, causing vomiting and acute “rice water” diarrhea, which can lead to deadly water loss of up to 15 L per day [4]. Cholera results from the absorption of *Vibrio cholera* contaminating water or food [5]. The WHO reports 1.3 to 4 million cases of cholera worldwide each year, including 21,000 to 143,000 deaths, making this disease a major public health problem in more than a third of countries, with 60% of cases in sub-Saharan Africa and 29% in Southeast Asia [5,6].

Cholera first appeared in Cameroon in 1971. Between 2010 and 2017, epidemiological surveillance recorded 37,396 cases with 1,646 deaths. The North and Far-North regions accounted for nearly 60% of all cholera cases during this period. The average case fatality rate was high (5.4%). Maroua in northern Cameroon is among the localities most frequently affected by cholera epidemics in the country [2,7]. The incidence of cholera in this city is influenced by several factors, including poor hygiene conditions and a low level of sanitation favorable to the development of *V. cholerae*.

*V. cholera* has important interactions with the environment and with humans [8]. Consequently, it interacts directly or indirectly with micro- and macroorganisms in its environment [9]. It interacts with chironomids during all four stages of the insects´ development, from egg to adult [10]. Chironomids are natural reservoirs of *V. cholera* [11]. Chironomids are the most ubiquitous and abundant group of macroinvertebrates and are the most abundant in terms of the number of species and individuals [12]. Chironomid larvae are found in all freshwater and terrestrial environments and are abundant in running water (torrents, streams, and rivers) and stagnant water (ponds, lakes, and rice paddies) [13]. Any organism acting as a reservoir of *V. cholera* contributes to its spread. A significant development of chironomids in the aquatic environment is favorable to the growth of *V. cholerae*; therefore, there may be a correlation between cholera outbreaks and the proliferation of chironomids [9].

In Africa, research on the natural reservoirs of *V. cholerae*, its vectors, hosts, and their mode of transmission to humans is rare [14]. In Cameroon, several studies have been carried out on *V. cholerae*, focusing on environmental reservoirs and factors influencing its survival and growth without specifically addressing chironomid larvae as potential reservoirs of *V. cholerae* [15,16]. Information on chironomid larvae in surface waters and their implication on the survival and growth of *V. cholerae* in cholera-endemic areas remains largely unknown. Chironomids are abundant in polluted water ecosystems and can potentially act as environmental reservoirs for *V. cholerae*, facilitating its persistence and dissemination in the cholera endemic region. Then, what is the density of chironomid larvae in the breeding sites of Maroua streams? What are the physicochemical characteristics of these aquatic environments? Do chironomid larvae of the Maroua streams carry *V. cholerae*? Is there a correlation between the presence of chironomid larvae and the persistence of *V. cholerae* in aquatic environments? This study aimed to determine the role that chironomid larvae may play in the maintenance and spread of *V. cholerae* in the water reservoirs of a cholera-endemic area in Cameroon.

## Methods

**Figure 1:**
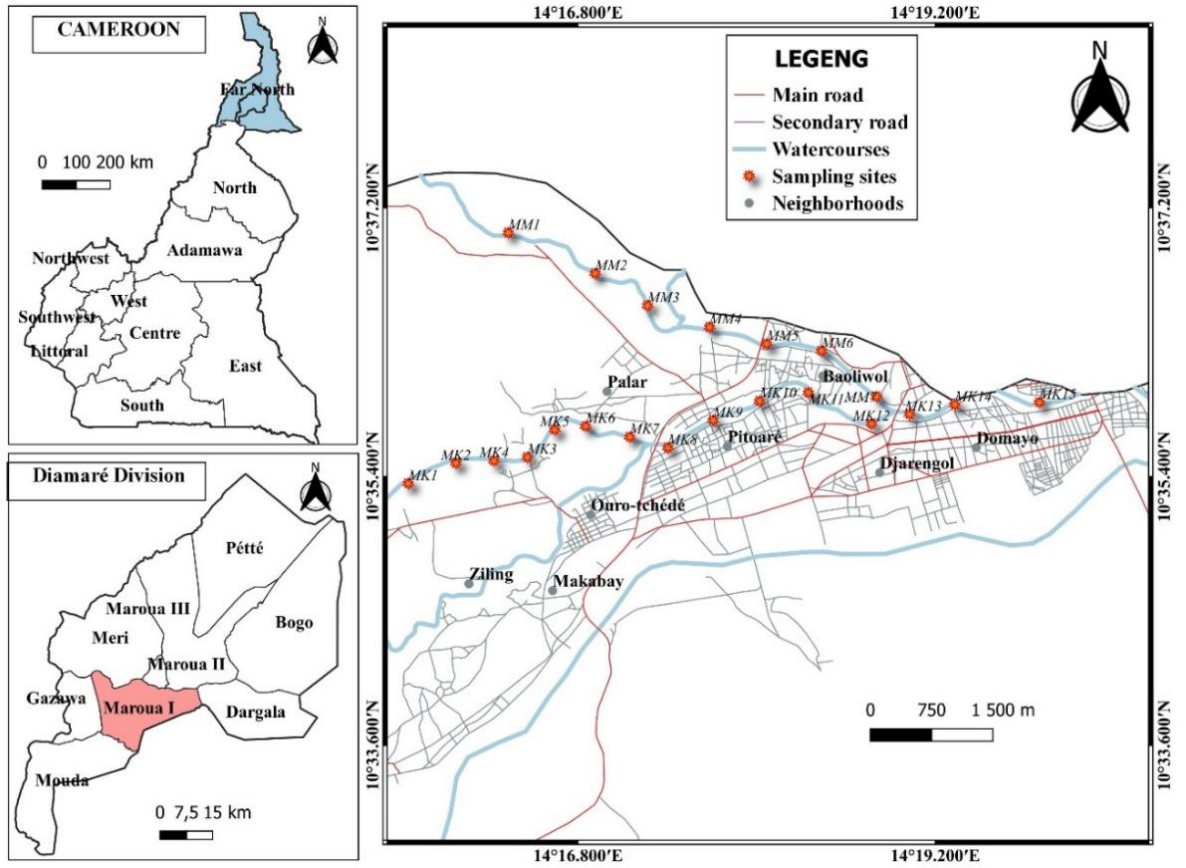
location of the study area and sampling sites on the map of the city of Maroua

**Study site:** Maroua is the capital town of the Far North Region, situated in the Diamaré Division. Maroua town is located between latitudes 10° 30' and 10° 50' North and longitudes 14° 10' and 14° 40' East, at an average altitude of 400 m. The city's topography varies very little, with an altitude difference of less than 20 m between the most extreme points [17]. Maroua's climate is of the Sudano-Sahelian type, characterized by the alternation of two seasons: a dry season from October to May and a rainy season from June to September [18]. Annual rainfall is approximately 800-900 mm [19]. The rainfall was more regular and abundant in July and August. Air temperatures fluctuate between 18°C in December and January, and 45°C in March and April. The annual average of this parameter is 27±1°C [20]. The city of Maroua has an area of 56 km^2^ and an estimated population of 319,941 people [21]. Most of the population has no access to drinking water or sanitation, exposing them to a high risk of diseases linked to unsanitary conditions. Maroua hosts the most affected cholera districts during the last epidemics [22]. The cholera outbreaks usually occur during the rainy season, and the dry season is known as the inter-epidemic period. The city of Maroua is crossed by two mayos, seasonal rivers that dry up in the dry season: the Mayo Kaliao and its tributary, the Mayo Mizao, and the Mayo Tsanaga (Figure 1) [17]. Mayo plays a crucial role in supplying local populations with water. During the dry season, when other sources dry up, local people use mayo to draw water by digging out the sand of the mayo bed. The hand-dug water holes are called scoop holes. These scoop holes are characterized by their shallow depth and their importance for aquatic living organisms adapting to the drying-up of the Mayo. Their water is used for many purposes: drinking, washing up, and watering livestock. They are also crucial sites for the development of insects such as chironomid larvae.

**Selection of sampling sites:** seasonal streams (Mayo) through the town of Maroua were explored for the collection of water and chironomid larvae. An exploration tour was conducted from upstream to downstream in each month, and larval sites were surveyed during the dry season in October and November. Two streams, Mayo Kaliao and Mayo Mizao, were selected for this study due to their frequent use by local communities and their established link with the spread of waterborne diseases such as cholera [23, 24]. Tsanaga Mayo, which did not show any chironomid larval breeding sites, was not selected. During this survey, larval sites consisting of scoop holes in the sand of the Mayo riverbed were marked to facilitate their location for larval collection; the size of the larvae (the different stages) was taken into account to sort the stage 4 larvae during collection (all sites presenting only stages 1, 2, and 3 larvae were eliminated) and the level of water in the breeding sites was also taken into account, since those with small quantities of water (which can dry up overnight) were not selected.

**Collection of water samples and chironomid larvae:** exploration of the Mayos led to the selection of 22 sites, including 15 on the Kaliao Mayo and 7 on its tributary, the Mizao Mayo. Some characteristics of the sampling sites were documented to better understand their interaction with the presence of chironomid larvae, such as anthropogenic activities, sediment deposition, and the presence of other organisms (Table 1). Water from each larval site was collected in two bottles: one sterile glass bottle and one polyethylene bottle. Water in a polyethylene bottle was used to measure the physicochemical parameters. The water sample in a sterile glass bottle was used for bacteriological analysis (isolation of *V. cholerae*) [23]. Stage 4 chironomid larvae are easily identifiable by their red color, because of their high hemoglobin content, and were collected using tweezers to lift individuals from sediment/substrate on the banks of water bodies [25]. The density of chironomid larvae in each breeding site was documented. The collected larvae were kept in sterile Falcon tubes labeled separately (five fourth-stage larvae per site). Water samples and chironomid larvae were transported to the laboratory for the isolation and identification of *V. cholerae*. Sampling was done bimonthly during the dry season from October to December 2022.

**Table 1:** some characteristics found around the sites and in the water of the breeding sites larvae

Sites	Anthropogenic activities	Sediment deposition	Type of sediment	Presence of other organisms
MK1	Laundry	+	Clay	-
MK2	Pieces of tissue	+	Sandy-clay	Larvae and adults of other insects
MK3	Plastic and footwear	+	Sandy	Algae
MK4	Detergent	+	Sandy	Algae
MK5	-	+	Sandy	Larvae and adults of other insects
MK6	Human faeces	+	Sandy	-
MK7	-	+	Clay	Insects
MK8	Laundry	+	Sandy	Larvae of other insects
MK9	Detergent	+	Clay	Insect of fish
MK10	-	+	Sandy	-
MK11	Pieces of bread, cardboard, cloth, and plastic	+	Sandy-clay	Algae et insects
MK12	Polyethylene bottle	+	Sandy	Larvae of other insects
MK13	Human faeces	+	Clay	-
MK14	-	+	Sandy	-
MK15	Human faeces	+	Sandy	-
MM1	Polyethylene bottle and beef droppings	+	Sandy	-
MM2	Laundry	+	Sandy	Larvae of other insects
MM3	Human faeces	+	Sandy	Algae
MM4	Wig	+	Sandy	Larvae of other insects
MM5	-	+	Sandy	-
MM6	Laundry food residues	+	Clay	Algae
MM7	Food residues and Pieces of tissue	+	Sandy	-

MK: Mayo Kaliao and MM: Mayo Mizao; -: absent; +: average; +: average accentuated; +: very accentuated

**Physicochemical analysis of water:** physicochemical water analysis was performed in situ using an Extech EC 500 multiparameter analyzer [23]. This device was used to measure four parameters: salinity, temperature, electrical conductivity, and total dissolved solids (TDS) content of the water samples. The Extech DO210 oximeter was used to measure the dissolved oxygen saturation rate (%) [26]. Titrimetric determination of ions was performed in the laboratory to determine the total hardness, magnesium, and calcium concentrations [27]. These parameters were chosen because of their importance to bacterial metabolism and the availability of laboratory equipment.

**Search for and isolation of *Vibrio cholerae* on chironomid larvae and in breeding water:** bacteriological analysis of water in sterile glass vials consisted of searching for, isolating, and identifying *V. cholerae*, which was enriched in alkaline water according to the protocol of Djaouda *et al*. (2020) [23]. To detect *V. cholerae* on chironomid larvae, larvae were crushed with a sterile glass rod in test tubes containing alkaline peptone water at pH 8, vortexed for 30 s, and incubated for 24 h at 37°C [25]. At the end of incubation, an inoculum of the culture broths seeded above was gently picked just above the surface using a platinum loop and streaked on Thiosulfate Citrate Bile Salt Sucrose (TCBS) agar (Liofilchem s.r.i. Bacteriology Products, Italy), and incubated for 24 h at 37°C. Presumptive colonies of *V. cholerae* (yellowish, 24 mm diameter) were subcultured on the surface of brain heart infusion agar (BHA) and incubated for 24 h at 37°C. Bacteria from the previous isolation techniques on BHA were subjected to orientation tests (microscopic observations, oxidase test, indole test, and Kligler Iron Agar tests) followed by identification tests on the API 20E kit tests, and the affinity of *V. cholerae* for NaCl (growth at concentrations between 0 and 6% NaCl) was checked. The results were read and interpreted with reference to an analytical catalogue [23].

**Data analysis:** the spatial variation in chironomid larvae density across breeding sites in Maroua streams was assessed, along with the physicochemical characteristics of the aquatic habitats and the spatiotemporal distribution of *Vibrio cholerae*. Larval densities per site and stream were quantified and visualized using bar charts. Key water quality parameters (e.g., temperature, pH, dissolved oxygen) were measured at each site, and their variability was similarly illustrated. Data normality was verified using the Shapiro-Wilk test before analysis. Differences in mean larval densities, physicochemical parameters, and *V. cholerae* counts across sites were evaluated using ANOVA, with Tukey´s post hoc test for pairwise comparisons where significant effects were detected (p< 0.05). The relationship between bacterial abundance in water and larval matrices was assessed using Spearman´s rank correlation, suitable for monotonic relationships. To explore multivariate relationships, Principal Component Analysis (PCA) was applied to reduce dimensionality and visualize correlations among *V. cholerae* presence, larval density, and physicochemical variables. Hierarchical Ascending Classification (HAC) further grouped sampling sites into clusters based on similar environmental profiles. All analyses were performed using R software (version 3.6.3; R Core Team).

## Results

### Variation of the density of chironomid larvae across the breeding sites

**Figure 2:**
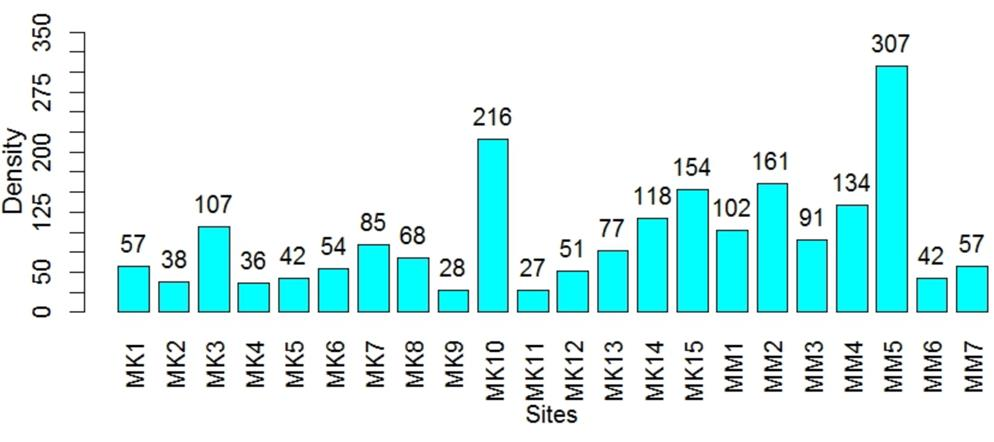
variation in chironomid larval density as a function of sampling sites

The density of chironomid larvae in this study ranged from 27 to 307 larvae. The lowest density was recorded at site MK11 (27 larvae) and the highest at site MK10 (216 larvae) among the 15 sampling sites in Mayo Kaliao. In Mayo Mizao, the lowest density was observed at site MM6 (42 larvae), and the highest density was observed at sampling site MM5 with 307 larvae (Figure 2). However, this variation in larval density with Mayo and sampling site location was not statistically significant as a function of Mayo and location (upstream, midstream, and downstream) (P>0.05).

### Physicochemical characteristics of breeding waters

Physicochemical characteristics determine the presence of *V. cholerae* in the aquatic environment. They varied from one sampling site to another and from one Mayo to another.

### Electrical conductivity

Electrical conductivity ranged from 147 to 530 µS/cm at all sites. The electrical conductivity varied between 147 (MK3) and 374 µS/cm (MK2) in Mayo Kaliao, and between 426 (MM6) and 530 µS/cm (MM7) in Mayo Mizao. It varied from one sampling site to another and from one Mayo to another. The lowest values for water electrical conductivity were observed in Mayo Kaliao, and the highest values were observed in Mayo Mizao (Annex 1, Figure 1). The electrical conductivity values were higher in Mayo Mizao than in Mayo Kaliao. The electrical conductivity values were used to classify the sampling sites into three (03) groups: - sites MK3 and MK4, forming group 1, have waters with low mineralization, with electrical conductivity between 100 and 200µS/cm: group 2 comprises sites (MK1, MK5, MK6, MK7, MK8, MK9, MK10, MK11, MK12, MK13, MK14 and MK15) characterized by an average mineralization between 200 and 333µS/cm; group 3 comprises sites (MK2, MM1, MM2, MM3, MM4, MM5, MM6 and MM7) with average mineralization between 333 and 666µS/cm. The two-way analysis of variance (ANOVA) test involving the Mayo and location sampling site (upstream, midstream, downstream) showed a significant difference in conductivity with the Mayo (Mayo: P<0.05; F= 6.24x10^-8^) and confirms an existing relationship between Mayo and mineralization level.

### Temperature

The temperature of the water sampled at the various sampling sites in the Kaliao Mayo fluctuated between 25.1 and 37.2°C (MK6 and MK5) and between 22.6 and 31.2°C (MM1 and MM7) in the waters of Mayo Mizao. Temperatures varied from one sampling site to another as well as from one mayo to another (Annex 1, Figure 1). There was a significant variation in temperature between the sampling sites and Mayo. ANOVA showed significant variations (P<0.05) in temperature as a function of Mayo and sampling site location (Mayo: P<0.05; F= 2.53x10^-5^; location: P<0.05; F= 0.0416).

### Total dissolved solids (TDS)

Concentrations of total dissolved solids fluctuated from one sampling site to another and from one Mayo to another. The lowest concentration of surface water TDS in Kaliao Mayo was 102mg/L (MK3), and the highest was 223 mg/L (MK1). As for the Mizao mayo, site MM4 recorded the lowest TDS concentration (274 mg/L) and MM7 the highest (318 mg/L). In general, the lowest TDS values were found in the waters of Mayo Kaliao and the highest values in Mayo Mizao. All TDS values in Mayo Mizao were higher than those in Mayo Kaliao (Annex 1, Figure 1). Statistical test results support the view that this variation is a function of Mayo (Mayo: P<0.05; F=2.04x10^-8^).

### Salinity

Surface water salinity values for the two Mayo (Kaliao and Mizao) varied according to the mayo. Salinity values fluctuated between 79.4 and 206 ppm (MK3 and MK10, respectively) in Kaliao Mayo and between 208 and 254 ppm in Mizao Mayo (MM2 and MM7, respectively). Overall, the lowest salinity values were observed in the Kaliao Mayo and the highest in the Mizao Mayo (Annex 1, Figure 1), while all Mizao Mayo sites had higher water salinity values than the Kaliao Mayo sites. This variation is Mayo-related (P<0.05; F= 5.19x10^-5^), as for the other physicochemical parameters.

### Dissolved oxygen saturation rate

Significant spatial fluctuations in dissolved oxygen saturation rate (TS-OD) were observed. Oxygen saturation levels in the Kaliao Mayo ranged from 42.8% to 91% (MK14 and MK6, respectively), and between 52% and 75.5% (MM1 and MM3, respectively) in the Mizao Mayo. The lowest oxygen saturation was recorded in Kaliao Mayo and the highest in Mizao Mayo (Annex 1, Figure 1). The TS-OD data collected enabled us to identify three groups of surface water sites (Mayo) according to the criteria defined by [23]: - MK1, MK2, MK3, MK4, MK9, MK12, MK14, MM1, and MM7 sites with low oxygen saturation (less than 60%); - sites MK5, MK7, MK11, MK15, MM2, MM3, MM4, MM5, and MM6 with acceptable oxygen levels (between 60 and 79%) for most aquatic organisms; -sites MK6, MK8, MK10, and MK13 with excellent oxygen levels for most aquatic organisms in the water (between 80 and 125%). This variation was unrelated to either the Mayo or the location (upstream, middle, and downstream) of the sampling sites (P>0.05).

### Calcium concentration

Calcium concentration varied between 20.03 and 180.31 mg/L (MK1/MK2/MK6/MK7 and MK4) in Kaliao Mayo and between 40.06 and 120.18 mg/L (MM6 and MM1) in the Mizao Mayo. The lowest and highest calcium hardness values were observed in Mayo Kaliao (Annex 1, Figure 1). Waters from sites MK1, MK2, MK3, MK6, MK7, MK8, MK9, MK10, MK11, MK12, MK13, MK14, MK15 of Mayo Kaliao and MM2, MM3, MM4, MM5, MM6 and MM7 (calcium between 20.03 and 100.15 mg/L) from Mayo Mizao have a low to medium calcium concentration and approximately equal to the maximum permissible concentration (100mg/L). Whereas water from sites MK4, MK5, and MM2 (between 120.18 and 180.31 mg/L) has exceeded the maximum permissible concentration of 100 mg/L. The site MK15 had a calcium concentration almost equal to the maximum concentration (100.15 mg/L). This oscillation in values is neither a function of Mayo nor of the location (upstream, midstream, and downstream) of the sites (P>0.05).

### Magnesium concentration

Water magnesium concentration fluctuated between 12.15 and 133.65 mg/L (MK15 and MK4/MK12) in Mayo Kaliao and between 12.15 and 145.80 mg/L (MM1 and MM7) in the waters of Mayo Mizao. These values varied from one larval site to another and from one may to another. The sites MK1, MK2, MK4, MK8, MK9, MK10, MK11, MK12, MM4, MM5, and MM7 (between 60.75 and 145.8 mg/L) exceeded the maximum magnesium concentration permitted for drinking water (50 mg/L). Conversely, sites MK3, MK5, MK6, MK7, MK13, MK14, MK15, MM1, MM2, MM3, and MM6 (between 12.15 and 48.6 mg/L) are within the range of the maximum magnesium concentration for drinking water (50 mg/L). Variations in Magnesium concentration as a function of Mayo were significant (P>0.05).

### Presence of *Vibrio cholera* in water and on chironomid larvae

*Vibrio cholerae* was isolated from water and chironomid larvae from Mayo Kaliao and its tributary Mayo Mizao (Table 2). *V. cholerae* strains were isolated and identified from 16 sampling sites. Only site MK6 contained isolates of *V. cholerae* in water and on chironomidae larvae; the rest of the strains were isolated either in water or on chironomid larvae. 27.72% (95% CI: 18.4-38.6) of water samples were positive for *V. cholerae*, where the bacterium was not isolated from chironomid larvae. Moreover, 4.54% (95% CI: 1.2-11.3) of water samples were positive for *V. cholerae*, where the bacterium was also isolated from the chironomid larvae. However, 45.45% (95% CI: 32.5-58.9) of breeding sites contained V. cholerae-positive chironomid larvae without the planktonic form of the bacterium in the water (Table 2). *V. cholerae* was detected in chironomid larvae at 11 sampling sites (MK1, MK2, MK3, MK6, MK7, MK9, MK13, MM1, MM4, MM6, and MM7) and water from 6 sampling sites (MK4, MK6, MK10, MK12, MK15, and MM2). Spearman's correlation test showed that the frequency of isolation of *V. cholerae* from chironomid is not related to larval density of the breeding site (P>0.05; rho= -0.1520862 and rho= 0.143182, respectively).

**Table 2:** number of *V. cholerae* strains isolated from water and chironomidae larvae, by sampling site

Samples sites	*V. cholerae* (water)	*V. cholerae* (larvae)
MK1	0	2
MK2	0	2
MK3	0	1
MK4	1	0
MK5	0	0
MK6	3	1
MK7	0	1
MK8	0	0
MK9	0	1
MK10	1	0
MK11	0	0
MK12	1	0
MK13	0	1
MK14	0	0
MK15	1	0
MM1	0	2
MM2	2	0
MM3	0	0
MM4	0	2
MM5	0	0
MM6	0	1
MM7	0	1

MK: Mayo Kaliao and MM: Mayo Mizao

### Relationship between *Vibrio cholerae* and breeding water characteristics

**Figure 3:**
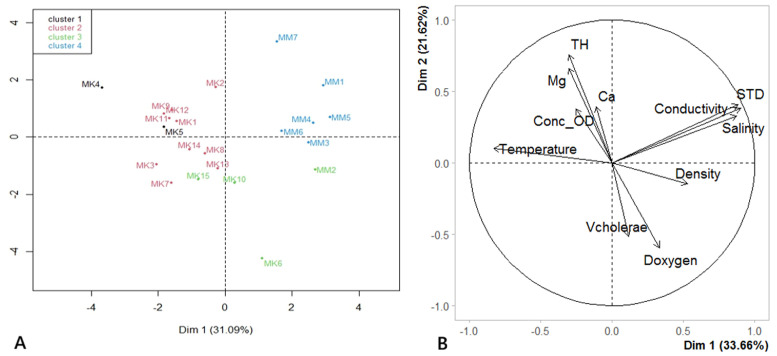
standardized PCA of physicochemical parameters and density: A) correlation circle of physicochemical parameters and *V. cholerae* isolated from water and larvae; numbers 1 to 22 represent sampling sites MK1 to MK15 (Mayo Kaliao) and MM1 to MM7 (Mayo Mizao); B) hierarchical ascending classification (HAC) plot of physicochemical parameters according to sampling sites

Principal component analysis (PCA) was used to assess the correlation between the frequency of isolation of *V. cholerae*, physicochemical parameters, and chironomid larvae density. Physicochemical parameters closely related to water mineralization (electrical conductivity, total dissolved solids (TDS), and salinity) were strongly positively correlated with each other. These parameters were also correlated with temperature, but in a negative direction on the axis of *V. cholerae* isolated from larvae (*V. cholerae*, 31.09% inertia) (Figure 3). When the temperature increased, the electrical conductivity, salinity, and STD decreased; conversely, an increase in electrical conductivity, salinity, and STD corresponded to a decrease in temperature, but had little influence on the presence of *V. cholera* on chironomid larvae. Total hardness and magnesium (Mg) were correlated with each other and with dissolved oxygen saturation, but also in the negative direction on the axis of *V. cholera* isolated from water (19.98% inertia). This is because an increase in the concentration of calcium and magnesium salts in water induces a decrease in the dissolved oxygen saturation level (TS-OD) and, consequently, the presence of *V. cholerae*. Low levels of oxygen saturation can inhibit *V. cholerae* growth. The density of chironomid larvae and dissolved oxygen content contributed little to the formation of axes 1 and 2 of the PCA compared to other chemical parameters (Figure 3). Some of these physicochemical characteristics correlated with the presence of *V. cholerae* in the water and on chironomid larvae, whereas others did not.

Figure 3 shows that the positions of the sampling sites remained constant from one Mayo to another in relation to the axes. Sampling sites influencing axis 1 are located close together (except for MK6 and MM7), for example, whereas when following axis 2, sampling sites MM1, MM2, MM3, MM4, and MM5 are almost all far from this axis. Sampling sites MK6 and MM7 did not move exactly along axis 1. The factors contributing to the variability in physicochemical characteristics did not appear to be the same for the different sampling sites.

## Discussion

### Density of chironomid larvae

Chironomids, recognized bioindicators of eutrophic conditions, exhibited no significant difference in larval density between the two studied Mayos (P> 0.05), suggesting comparable anthropogenic pressures in both systems. However, within-site variations in density may reflect localized differences in contaminant loads, interspecific competition, and sediment composition-factors previously identified as critical drivers of chironomid distribution [13,28]. These findings align with broader ecological studies emphasizing the sensitivity of chironomids to microhabitat conditions, particularly in anthropogenically impacted freshwater ecosystems.

### Physicochemical characteristics of sampled breeding waters

The physicochemical analysis revealed pronounced spatial heterogeneity in water properties, particularly in electrical conductivity (147-530 µS/cm), which varied significantly between mayos (P < 0.05). Mayo Kaliao exhibited a gradient from low to high mineralization (147-374 µS/cm), whereas Mayo Mizao consistently showed medium-high mineralization (426-530 µS/cm). This disparity likely stems from differences in soil composition and proximity to anthropogenic inputs, consistent with observations by Mapoma *et al*. (2017) and Ayad *et al*. (2016) [29,30]. Notably, the correlation between conductivity and localized pollution sources underscores the role of human activities in shaping water chemistry, a pattern documented in similar tropical systems [31,32].

Temperature fluctuations (25.1-37.2°C) further highlighted microenvironmental variability, with significant differences by Mayo and sampling location (P< 0.05). These ranges are conducive to microbial activity, including Vibrio spp., whose survival is temperature-dependent [33,34]. Similarly, dissolved oxygen saturation (42.8-91%) and hardness parameters (Ca^2+^: 20.03-180.31 mg/L; Mg^2+^: 12.15-145.80 mg/L) reflected site-specific influences from organic matter degradation and geological inputs, respectively. Elevated hardness at certain sites (>100 mg/L Ca^2+^; >50 mg/L Mg^2+^) exceeded recommended limits for drinking water [35], suggesting potential anthropogenic contributions [36].

### Association of *V. cholera* with chironomid larvae

*V. cholerae* was detected in 72.7% of sampling sites (16/22), with a higher prevalence on chironomid larvae (50% of sites) than in water (27.3%). This reinforces the well-documented symbiosis between *Vibrio* and aquatic invertebrates [10,11]. Chironomid larvae, in particular, appear to serve as reservoirs for toxigenic strains (e.g. O1/O139), as noted by Laviad *et al*. [25]. Their role in *V. cholerae* ecology is critical, as larval abundance and distribution may directly influence pathogen dynamics in cholera-endemic regions.

### Physicochemical drivers of *Vibrio cholerae* occurrence

The prevalence of *V. cholerae* was strongly associated with dissolved oxygen saturation, total hardness, and magnesium hardness. Higher oxygen levels correlated with increased bacterial concentrations, supporting earlier findings that aerobic conditions favor *V. cholerae* proliferation [37,38]. Conversely, elevated hardness (particularly Mg^2+^) appeared inhibitory, likely by disrupting ion-dependent cellular processes [39]. This aligns with studies demonstrating Mg^2+^ toxicity at concentrations >100 mg/L, which impair bacterial viability and metabolic activity [40]. Such thresholds were observed at several sites (e.g., MM7: 145.80 mg/L Mg^2+^), suggesting that water chemistry may locally modulate *V. cholerae* persistence.

This study highlights the interplay between physicochemical factors, chironomid ecology, and *V. cholerae* persistence in aquatic systems of a cholera-endemic region of Cameroon. Future research should explore the seasonal dynamics of *V. cholerae*-chironomid interactions and the efficacy of hardness-based mitigation strategies in cholera-prone areas.

## Conclusion

Chironomid larvae and the water in which they live are reservoirs of *V. cholerae*. This study shows that in the aquatic environment, *V. cholerae* is more closely associated with chironomid larvae (potential reservoirs) rather than freely in the water to ensure its survival and growth. Dissolved oxygen saturation levels, total hardness, and magnesium hardness also played a decisive role in the survival and growth of *V. cholerae* in larval site waters. Variations in physicochemical parameters depend on the type of soil through which mayowater flows, pollutant inputs from human activities, and the sampling period. The density of chironomid larvae in surface waters is not to be overlooked, as *V. cholerae* colonizes chironomid larvae, which are reported to be their reservoirs.

### What is known about this topic

- Cholera is a serious public health problem;
- Water reservoir of V. cholerae in the city of Maroua;
- Factors affecting the persistence of the cholera epidemic in North Cameroon.

### What this study adds

- Chironomids act as sentinels for both eutrophication and V. cholerae presence, underscoring their utility in environmental monitoring;
- Magnesium hardness and dissolved oxygen are critical modulators of V. cholerae survival, with potential implications for water quality management.

## Annex

**Annex 1**: variation in physicochemical parameters according to sampling sites: A) electrical conductivity, B) temperature, C) total dissolved solids, D) salinity, E) dissolved oxygen saturation (%), F) calcium hardness, G) magnesium hardness; (MK: Mayo Kaliao and MM: Mayo Mizao) (PDF - 389KB)
